# High-Quality Draft Single-Cell Genome Sequences of Two *Gammaproteobacteria* Strains Sampled from Soil in a Strawberry Farm

**DOI:** 10.1128/MRA.00743-20

**Published:** 2020-08-27

**Authors:** Takuya Yoda, Koji Arikawa, Tatsuya Saeki, Ayumi Matsuhashi, Masahito Hosokawa

**Affiliations:** a bitBiome, Inc., Tokyo, Japan; University of Southern California

## Abstract

Here, we present high-quality draft single-cell genome sequences of *Gammaproteobacteria* strains BBSC-SA01 and BBSC-SA02, obtained from uncultivated cells of soil in a strawberry farm using the single-cell sequencing platform bit-MAP. These draft genomes putatively represent novel species within *Gammaproteobacteria* and allow further investigation into the soil microbiome.

## ANNOUNCEMENT

*Gammaproteobacteria* is a class of Gram-negative bacteria comprising 17 orders and plays an important role in crop and animal health in various soil microbiomes (1, 2). Although their universal distribution and prevalence in the soil environment are revealed by 16S rRNA gene amplicon sequencing and shotgun metagenomic sequencing, most of them remain uncultivated at this time (3). Therefore, an expansion of the reference genomes of *Gammaproteobacteria* would lead to an improved understanding of their genetic diversity and relationship with the environment and other microbial species. In our present study, we sequenced two uncultivated strains of *Gammaproteobacteria* using the single-cell genome sequencing platform bit-MAP, originally named SAG-gel (4).

Soil sampling was conducted in two strawberry cultivation greenhouses before harvest in December 2019 (Okawa, Fukuoka, Japan). A total of 20 g of soil sample was collected at a depth of 5 to 10 cm around the seedlings. To extract the bacterial fraction, 1.5 g of the soil samples was suspended in 3 ml of Dulbecco’s phosphate-buffered saline (DPBS). The mixture was vortexed and allowed to settle for 15 min and then filtered to separate the soil particles. Single cells were isolated into gel beads, and their genomes were amplified using the REPLI-g kit (Qiagen) following the SAG-gel method (4). Single gel beads harboring a single-cell amplified genome (SAG) were sorted into individual wells in a 96-well microplate (Axygen) using BD FACSMelody (BD Biosciences) and then reamplified with the REPLI-g kit. A pooled paired-end sequence library (300 × 2 bp) of 24 SAGs was prepared using the Nextera XT DNA sample preparation kit (Illumina) and sequenced using a MiSeq sequencer (Illumina). Default settings were used for data analysis unless otherwise noted. Sequence reads were filtered using BBduk v38.79 (https://sourceforge.net/projects/bbmap/) with the parameters qtrim=r, trimq=10, minlength=40, maxns=1, and minavgquality=15; they were assembled with SPAdes v3.14.0 (5) using the settings –t 4, --sc, --careful, and --disable-rr, and contigs of >1,000 bp were retained for the following analysis. Completeness and contamination were calculated using CheckM v1.1.2 (6) using the option --reduced_tree. The number of contigs, *N*_50_ value, and total length of SAGs were evaluated with QUAST v5.0.2 (7). The number of tRNAs was examined with Prokka v1.14.6 (8) using the option --mincontiglen 200. The 16S rRNA gene sequence was extracted with Prokka and assigned to RefSeq with a BLASTn v2.9.0+ search (9). The SAGs were taxonomically classified with GTDB-Tk v1.1.1 (10), and the average nucleotide identities (ANI) were calculated with sendsketch.sh v38.79 (https://www.biostars.org/p/234837/) against the RefSeq genome database (NCBI).

The genome statistics are given in Table 1. Based on the operational standards for SAGs (11), they are classified as high-quality draft genomes. They were classified as *Legionellales* bacterium UBA4722 (strain BBSC-SA01) and *Gammaproteobacteria* bacterium RIFCSPHIGHO2_12_FULL_45_12 (strain BBSC-SA02) with GTDB-tk. Comparison with 16S rRNA gene sequences using RefSeq found the two most closely related 16S rRNA sequences to be Legionella dresdenensis strain W03-356 (GenBank accession number NR_115062.1, 88.38%) and Aquicella siphonis strain SGT-108 (NR_025764.1, 94.553%), respectively. With respect to the maximum ANI among all available RefSeq genomes, the highest similarities of BBSC-SA01 and BBSC-SA02 were observed with Methylophaga sulfidovorans (73.4%) and Aquicella lusitana (78.2%), respectively. The ANI between the two genomes was only 63.4%, suggesting that BBSC-SA01 and BBSC-SA02 were distantly related to each other.

**TABLE 1:** Genome statistics of BBSC-SA01 and BBSC-SA02

Statistic	Data for strain:	
	BBSC-SA01	BBSC-SA02
No. of reads	534,470	561,038
Genome size (bp)	2,056,922	2,101,049
No. of contigs	147	229
*N*_50_ (bp)	39,139	41,260
G+C content (%)	39.4	37.5
Completeness (%)	92.73	91.53
Contamination (%)	0.68	1.16
Total no. of genes	1,890	1,964
No. of tRNAs	40	40
No. of rRNAs (5S, 16S, 23S)	3 (1, 1, 1)	3 (1, 1, 1)
Coverage (×)	140	133
Genome GenBank accession no.	BMAG00000000	BMAH00000000
SRA no.	DRR236341	DRR236342

### Data availability.

The genome sequences reported in this article were deposited in DDBJ/ENA/GenBank under BioProject number PRJDB10155 and the accession numbers given in Table 1. The versions described in this paper are the first versions.
